# Discovery and computational studies of piperidine/piperazine-based compounds endowed with sigma receptor affinity†

**DOI:** 10.1039/d3md00291h

**Published:** 2023-07-26

**Authors:** Laura De Luca, Lisa Lombardo, Salvatore Mirabile, Agostino Marrazzo, Maria Dichiara, Giuseppe Cosentino, Emanuele Amata, Rosaria Gitto

**Affiliations:** a Dipartimento di Scienze Chimiche, Biologiche, Farmaceutiche ed Ambientali, Università degli Studi di Messina Viale Ferdinando d'Alcontres 31 98166 Messina Italy rgitto@unime.it; b Dipartimento di Scienze del Farmaco e della Salute, Università degli Studi di Catania Viale Andrea Doria 6 95125 Catania Italy

## Abstract

Herein, we describe our efforts to identify sigma receptor 1 (S1R) ligands through a screening campaign on our in-house collection of piperidine/piperazine-based compounds. Our investigations led to the discovery of the potent compound 2-[4-(benzyl)-1-piperidin-1-yl]-1-4-(4-phenylpiperazin-1-yl)ethanone (1) with high affinity toward S1R (*K*_i_ value of 3.2 nM) that was comparable to reference compound haloperidol (*K*_i_ value of 2.5 nM). Functional assay revealed that compound 1 acted as S1R agonist. To decipher the binding mode of this promising S1R ligand as a starting point for further structure-based optimization, we analysed the docking pose by using a S1R-structure derived from cocrystal structures of potent ligands in complex with target protein. The computational study was enriched with molecular dynamic simulations that revealed the crucial amino acid residues that interacted with the most interesting compound 1.

## Abstract 2

Discovery of potent S1R agonist by means of screening campaign and analysis of binding interactions.

## Graphical abstract

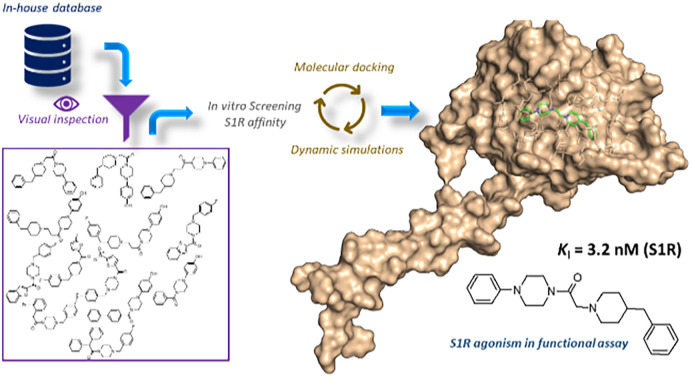

## Introduction

In recent years, significant efforts have been addressed toward drugs effective in modulating canonical targets *via* innovative pathways. Among them, sigma receptors (SRs) drew high interest in developing agents for different therapeutic areas.^1,2^ Indeed, the two SR subtypes sigma-1 (S1R) and sigma-2 (S2R) are involved in a large array of biological functions due to their ability to interact with various proteins and ion channels. They are assumed to modulate multiple signalling pathways; therefore, they might be considered valuable tools to identify innovative drugs for treatment of human diseases such as neurodegenerative pathologies, neuropsychiatric disorders, cancer, and so on.^1–12^ Crystal structures of human S1R and bovine S2R provided structural information for both receptor subtypes.^13–15^ S1R possesses a trimeric organization containing three distinct protomers, in which each protomer comprises a single transmembrane domain linked to four alpha-helices and one beta-barrel region for binding pocket of ligands. The crystal structure of bovine S2R is assembled as a transmembrane homodimer, that displays a four-helix bundle fold and contains the binding pocket localized in the centre of the protein. Before the release of cocrystal structure of S1R in complex with potent ligands, the S1R pharmacophoric hypotheses were based on classical structural-affinity relationship (SAR) evidence. The first pharmacophore model was developed by Glennon and co-workers that provided the suggestion that two hydrophobic pockets are linked through a central basic core as positive ionizable group.^16^ The optimal distance between hydrophobic features (called primary and secondary hydrophobic sites) characterizes potent and selective S1R ligands.^17^ Several small molecules have claimed as potent and selective ligands targeting S1R for the therapeutic area of central nervous system (CNS) drugs; on the other hand, the S2R ligands are characterized by two hydrophobic features and an amine basic centre.^18,19^ Moreover, structural data provided evidence that S1R and S2R share similar amino acid residues that are considered relevant for binding interactions with potent ligands; therefore, there is a large series of mixed S1R/S2R ligands showing the ability to exert antiproliferative effects as anticancer agents.^19^ Despite there is a large collection of small molecules possessing the capability to bind S1R and/or S2R, the identification of new ligands could offer additional information to reveal the features that might play essential role in subtype selectivity.^20^

It is well-known that the quest from “hit compounds” to “lead candidates” is often characterized by a long process in designing newer chemical entities and arduous synthetic efforts in obtaining sufficient number of compounds for preliminary screening thus collecting SARs. Among the medicinal chemistry strategies to optimize the drug discovery process, a fruitful approach foresees the screening campaign of in-house database of already synthesized compounds to carry out preliminary biochemical testing. As a result, new hit compounds might be identified through a further process of structural optimization in terms of improvement of potency and selectivity toward selected molecular targets. This very simple approach became from the best knowledge and intuitive capabilities of the medicinal chemist to select the best “hit candidates” in the existing libraries of compounds. Considering the above, in the present work we have selected thirteen compounds among our previously reported small molecules to measure their affinity at S1R and S2R, as this small series of arylpiperazine-based compounds could possess the minimal structural requirements for binding recognition to S1R and/or S2R as drawn in canonical representation of pharmacophoric models reported in literature.^3,20^ The S1R and S2R affinities, functional assay and docking simulations were applied to investigate the mode of action of this new series of potential sigma ligands.

## Results and discussion

### Compounds selection

**Fig. 1:**
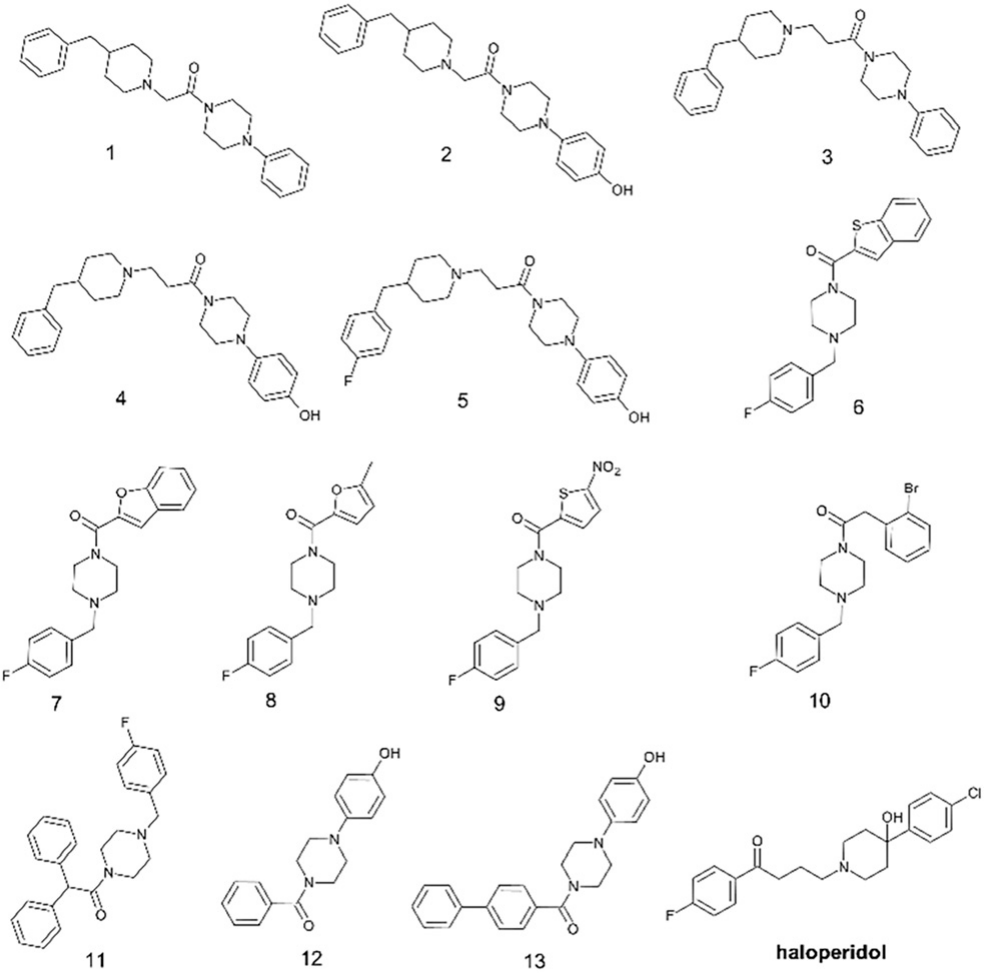
Chemical structures of selected aryl/arylmethyl-based compounds 1–13 extracted from our in-house database of synthesized compounds and the reference compound haloperidol.

In the present work we have selected thirteen compounds 1–13 among our previously reported small molecules to measure their affinity at S1R and S2R. The chemical structures of studied compounds 1–13 are displayed in Fig. 1, which also reports the chemical structure of haloperidol as prototype of S1R/S2R ligands. In detail, eleven of thirteen compounds contain a cyclic amine moiety as basic core of the molecule; additionally, compounds 12 and 13 lacking this feature were also considered to confirm the role of positive ionizable moiety as relevant pharmacophoric element. Like to haloperidol (Fig. 1) this set of thirteen compounds also possess two aromatic rings combined with a variable linking group and additional features capable to create polar or halogen additional interactions with the sub-pockets characterizing each SR ligand binding site.

### Assessment of S1R and S2R affinity and structure–activity relationship (SAR) analysis

A screening of the selected compounds 1–13 was performed to measure the S1R and S2R affinity by means of the radioligand binding assay; the *K*_i_ values are collected in Table 1 and compared with reference compound haloperidol. For all compounds we *in silico* estimated the most relevant physicochemical properties to predict (a) their ability to passively permeate the biological membranes and (b) their ability to interact with SRs *via* protonated amine of the piperidine/piperazine ring (see Table 1).

**Table 1:** Binding affinity of compounds 1–13 and haloperidol toward S1R and S2R and their estimated physico-chemical properties

Cpd	*K*_i_ ± SD^a^ (nM)		clog *P*^b^	cp*K*_a_^b^	*N* ^+^ %
	S1R	S2R			pH = 7.4^c^
1	3.2 ± 0.7	104 ± 15	4.16	8.03	81.01
2	24 ± 5.0	1199 ± 226	3.42	8.03	81.01
3	8.9 ± 1.3	234 ± 43	4.33	9.46	99.12
4	328 ± 58	1002 ± 203	3.59	9.40	98.91
5	434 ± 47	610 ± 146	3.64	9.37	98.85
6	82 ± 18	>10 000	4.03	6.29	7.24
7	132 ± 27	>10 000	2.20	6.42	9.44
8	214 ± 29	>10 000	2.37	6.46	10.37
9	309 ± 55	>10 000	2.19	6.02	3.99
10	366 ± 80	>10 000	3.69	6.86	22.25
11	390 ± 77	>10 000	4.58	6.83	21.23
12	>10 000	>10 000	0.90	4.35	0.09
13	>10 000	>10 000	2.55	4.35	0.09
**Haloperidol** d	2.6 ± 0.4	77 ± 18	4.30	8.20	86.19

a Each value is the mean ± SD of at least two experiments performed in duplicate; S1R assay performed with [^3^H](+)pentazocine, S2R assay performed with [^3^H]DTG.

b Estimation of log *P* with ACD Lab estimation of p*K*_a_ with Marvin Sketch.

c Estimation of protonation states with Marvin Sketch version 22.21.0.

d Ref. 21.

The compounds 1–11 showed nanomolar affinity at S1R with *K*_i_ values ranging from 3.2 to 434 nM; compounds 1–5 showed lower affinity to S2R when compared to S1R, whereas compounds 6–11 exhibited no affinity toward S2R. Compounds 12 and 13 failed to bind both receptor subtypes. The binding results suggested that the basic amino moiety drives the S1R/S2R affinity and selectivity. Keeping in mind the critical role of the positively charged nitrogen atom to efficiently bind S1R, we calculated the p*K*_a_ of studied compounds to describe the percentage of protonated form for each molecule using MarvinSketch predictor (22.21.0 software version). The calculation of percentage of monoprotonated forms in an aqueous solution are reported in Table 1. This calculation revealed that only compounds 1–5 resulted largely in ionized form at physiological pH (>80% of positively charged nitrogen atom); in more details the benzylpiperidine derivatives 3–5 are present almost exclusively in the monoprotonated form at physiological pH in coherence with their highest basicity, whereas the two benzylpiperidine derivatives 1, and 2 possess a decrease of estimated basicity (p*K*_a_ of 8.03) and 81.01% of monoprotonated state. For remaining compounds 6–11 the p*K*_a_ predictions provided information about their poor ability to generate protonated forms at physiological pH. As expected, the proton addition to the nitrogen atom of the two derivatives 12 and 13 did not occur.

The protonation states of compounds 1–5 were in good agreement with their ability to bind both S1R and S2R, as found for reference compound haloperidol. Compounds 1 and 3 proved to be the more potent ligands for S1R (*K*_i_ of 3.2 and 8.9 nM, respectively) and exhibited a moderate selectivity over S2R (*K*_i_S2R__/*K*_i_S1R__ ratio 33 and 26, respectively); the best ligand 1 was able to interact with S1R at similar concentration of reference compound haloperidol (*K*_i_ of 2.6 nM). In the case of the best ligands 1 and 3, there is no evident influence of the length of alkyl linker. Interestingly, compound 2 showed good affinity for S1R (*K*_i_ of 24 nM) and improved selectivity over S2R (*K*_i_S2R__/*K*_i_S1R__ ratio 50). Based on the *K*_i_ values of compounds 1 and 3 when compared to analogue compounds 2 and 4, we observed that the 4-hydroxylphenyl-moiety was generally detrimental on the affinity for both S1R and S2R. Whereas, the 4-fluorophenyl-substituted derivative 5 resulted the poorer S1R ligand among the benzylpiperidine-derived compounds 1–5. Notably, the low basicity of benzylpiperazine-derived compounds 6–11 could explain their moderate S1R affinity combined with relevant selectivity over S2R (*K*_i_ > 10 000 nM) as found in literature for S1RA and PRE-084.^20^ Among this series, the best ligands 6–7 more efficiently interacted with S1R (*K*_i_ of 82 nM and 132 nM) to respect other congeners 8–11 possessing *K*_i_ values ranging from 214 to 390 nM thus revealing that there is significant influence of the nature of (hetero)aromatic moieties as crucial requirements to occupy the hydrophobic pocket. In accordance with previous evidence on the role of the presence of protonated amine, the poor basic compounds 12, and 13 did not bind to both S1R and S2R. Overall, among the tested compounds it was found a significant difference of calculated log *P* (clog *P*) values; the best active ligands proved to possess acceptable properties in membrane permeability.

### S1R functional assay for ligand 1

**Fig. 2:**
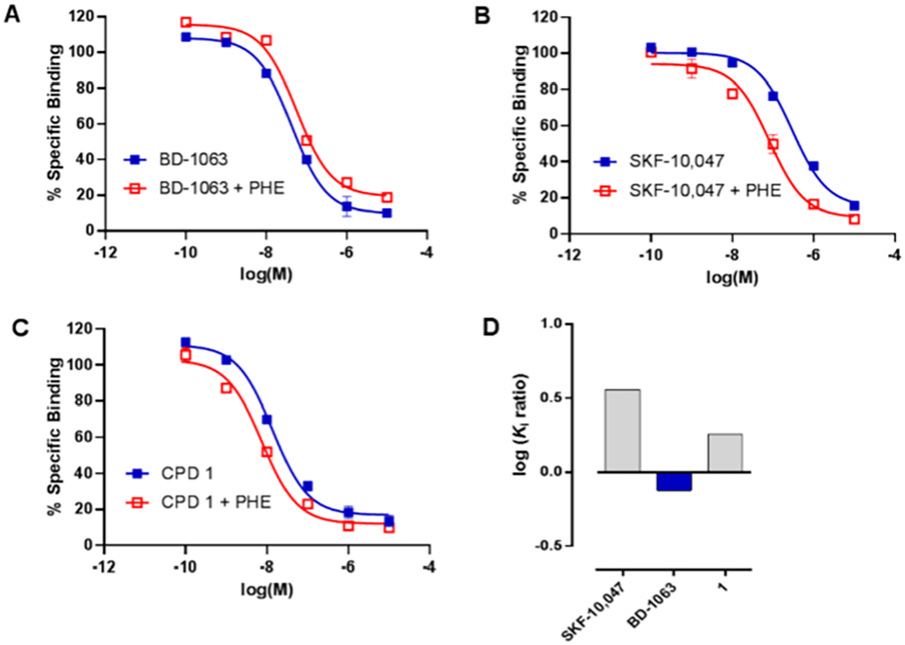
[^3^H](+)-Pentazocine displacement of (A) BD-1063, (B) SKF 10 047, and (C) compound 1 in the presence (red) or absence (blue) of phenytoin (PHE). Ratio of log *K*_i_ values with or without phenytoin (D) in the S1R binding assay.

Compound 1 was then subjected to *in vitro* phenytoin assay for S1R functional profile determination. Phenytoin is an allosteric modulator for the S1R, and it differentially modulates the affinity of S1R ligands on the basis of their agonist or antagonist profile.^22,23^ Indeed, phenytoin potentiates the receptor binding affinity of S1R agonists and produces no effects or slightly reduced receptor binding affinity for S1R antagonists. The functionality of compound 1 on S1R was determined by radioligand binding assay using rat liver in the presence of phenytoin, together with the known S1R agonist SKF-10 047 and antagonist BD-1063 (Fig. 2).

Compound 1 and SKF-10 047 showed a ratio of *K*_i_ without phenytoin/with phenytoin of 1.8 and 3.2, respectively. The reference compound BD-1063 exhibited a very small shift of the displacement curve with a ratio of 0.8. These observations^24^ indicate that compound 1 acts as a S1R agonist.

### Molecular modeling

**Fig. 3:**
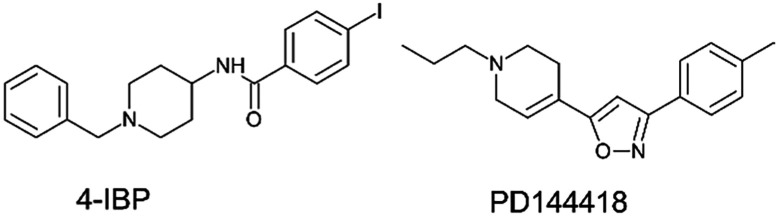
Chemical structures of the S1R ligands 4-IPB, and PD1444418.^3,20^

To streamline the experimental data collected for promising compounds 1–11 and to decipher their binding mode we performed a computational workflow including docking and dynamic simulations based on the available crystal structure of S1R in complex with the ligand 4-IBP (PDB code 5HK2).^13^ We chose to focus our interest on S1R for which we measured the best activity and selectivity. Our studies started with the protocol validation of the X-ray complexes of the above mentioned co-crystal structure to confirm the accuracy of our study. Additionally, the two crystal structures of well-known S1R ligands haloperidol and PD144418 were considered by analysing the two binary complexes 6DJZ^25^ and 5HK1 (ref. 13) to further enrich our computational procedure. The chemical structures of studied ligands haloperidol, 4-IPB and PD1444418 are represented in Fig. 1 and 3. More detailed information about the protocol validation can be found in the ESI.† Both the accuracy and the predictivity of our procedure were confirmed, then, this protocol was applied to the selected compounds 1–11 displaying different affinities determined by competition binding experiments on S1R (*K*_i_ values ranging from 3.2 to 434 nM) (Table 1). Based on the calculation of p*K*_a_ collected in Table 1, the molecular docking studies were performed considering the estimated prevalence of ionized or neutral form at physiological pH as calculated by using MarvinSketch predictor (22.21.0 software version).

In the first step, we ran a rigid docking employing the tool Glide.^26^ Therefore, the best Glide Emodel pose was submitted to a redocking calculation considering the flexibility of the side chain by means the Induced Fit Docking (IFD) tool of the Schrödinger Suite.^27^ Subsequently, the best Glide Gscore pose for each ligand was analyzed to evaluate the binding mode and the interaction in S1R site in comparison with well-known potent ligands 4-IBP, PD144418 and haloperidol. These data were employed to rationalize the computational data collected on congeners 1–5.

**Fig. 4:**
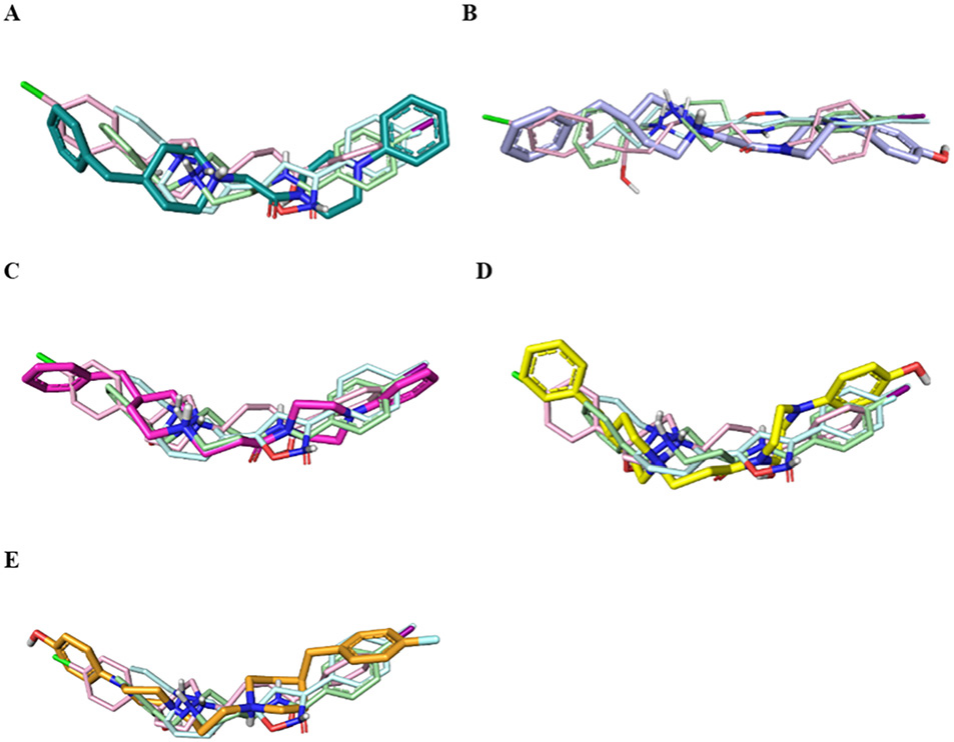
Superimposition of the co-crystalized ligands 4-IBP (pale green), PD144418 (pale cyan), and haloperidol (pale rose) aligned with compound 1 (deep teal, panel A), compound 2 (violet, panel B), compound 3 (hot pink, panel C), ligand 4 (yellow, panel D) and ligand 5 (orange, panel E).

Fig. 4 displays the predicted binding mode of each derivative 1–4 (panels A–D) in comparison with the crystallographic poses of 4-IBP (colored in pale green), PD144418 (colored in pale cyan) and haloperidol (colored in pale rose). As can be noted in the Fig. 4, the S1R ligands shared a similar binding pose assuming a linear arrangement in the binding site. Immediately, it was noted that they fruitfully occupied the central core as well as the two hydrophobic binding pockets characterizing the pharmacophore models^28^ as found for the crystalized ligands 4-IBP, PD144418 and haloperidol.^13,25^ Specifically, the piperidine nitrogen atom represented the positive ionizable functionality, the 4-phenylpiperazine tail efficiently assumed primary hydrophobic group and the benzyl moiety represented the secondary hydrophobic group. Compound 5 (Fig. 4E) lose the superimposition of the basic center when compared with the crystallized ligands; of note that compound 5 showed a reverse orientation with a 180° horizontal flip when compared to congeners 1–4 as well as ligands 4-IBP, PD144418 and haloperidol (Fig. 4A–D).

**Fig. 5:**
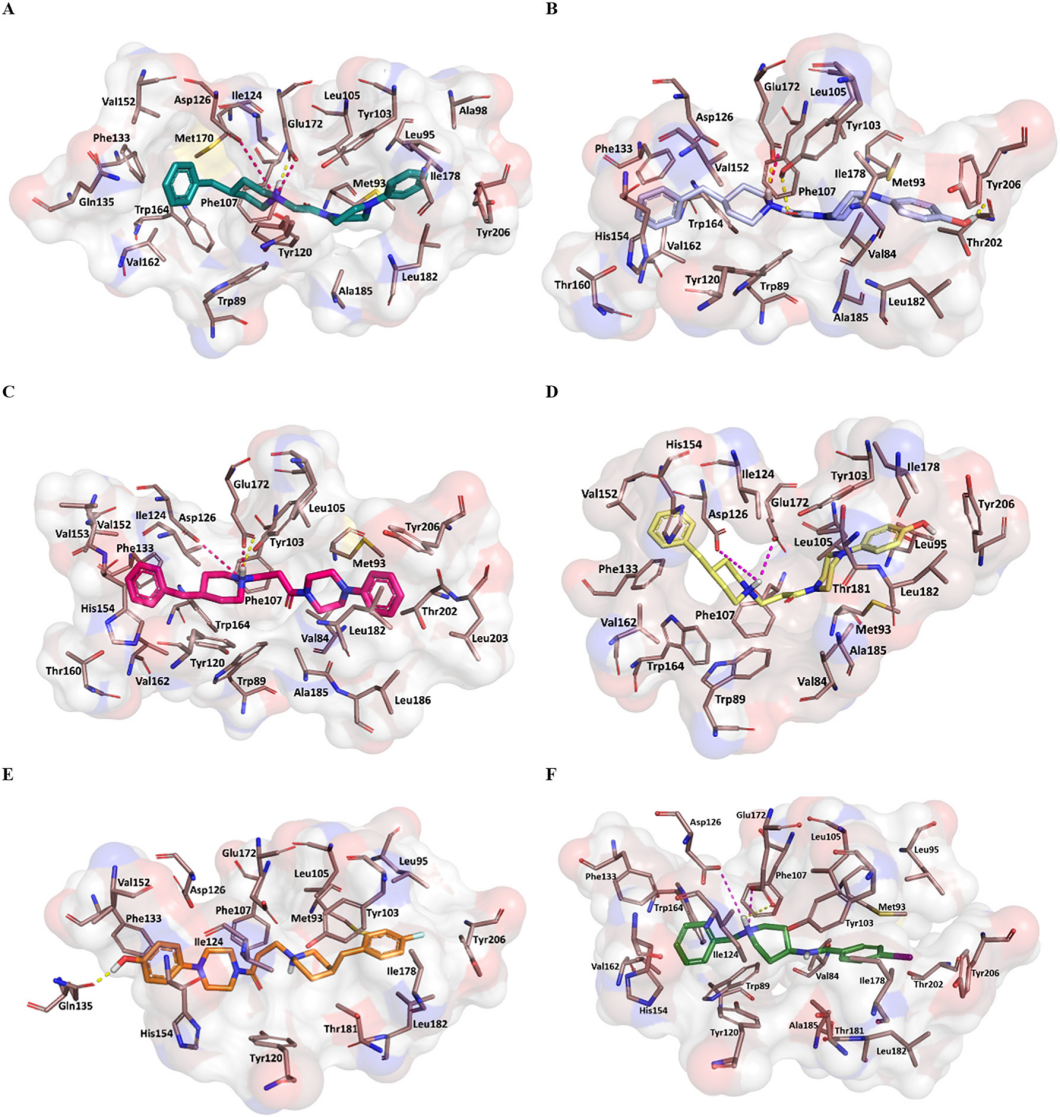
Plausible binding mode of compounds 1 (A) (deep teal stick), 2 (B) (violet stick), 3 (C) (hot pink stick), 4 (D) (yellow stick), 5 (E) (orange stick) and 4-IBP (F) (green stick) in the cavity of S1R. The residues of the binding site involved in the interactions with the ligands are displayed as dirty-violet sticks. For the residues forming hydrophobic interactions the surface is highlighted. The ionic and hydrogen bond is represented respectively as magenta and yellow dashes.

Fig. 5 reports the docked poses of studied ligands 1–5. In details, ligands 1 (panel A), 3 (panel C), and 4 (panel D) formed a bidentate salt bridge interaction involving the piperidine nitrogen atom and carboxylate groups of the Glu172 and Asp126 residues; for compound 2 (panel B) this polar interaction involved only Glu172. Notably, the high affinity ligands 1–3 established a hydrogen bond interaction with the side chain of Glu172. Moreover, compounds 1–4 were stabilized by a π–cation interaction between the ionized nitrogen atom and Phe107 residue. The hydrophobic residues Val84, Trp89 Met93, Leu95, Tyr103 Leu105, Ile178, Leu182, Leu203, Thr202, Tyr206 lined the primary hydrophobic region and were able to stabilize the ligands through van der Waals interactions. The network of amino acids Ile124, Phe133, Val152, His154, Val162, Trp164 formed the secondary sub-pocket and were involved in the hydrophobic contacts (Fig. 5A–D) with studied ligands. The binding mode of compound 4 suggests that the 4-hydroxyphenyl moiety would clash with aromatic ring of Tyr206. This evidence provided the consideration that the lengthening of linking group from two to three carbon atoms induced a peculiar pose for which the hydroxyl group was excessively close to a residue outlining the hydrophobic pocket thus generating a dramatic reduction of affinity of compound 4 when compared with shorter analogue 2. An even more significant reduction of affinity was observed for the weak 4-fluorophenyl-derivative 5 (Fig. 5E), which assumed a changed orientation when compared to unsubstituted analogue 4 (Fig. 5D). Therefore, the presence of 4-fluoro-substituent resulted in a different arrangement of compound 5; the positive ionizable feature was moved away from the Glu172 and Asp126 residues thus preventing the formation of the crucial ionic interactions; this hypothesis was in good agreement with its weak affinity (see Table 1).

Overall, the docking poses of the best ligands 1 and 3 revealed that they were strongly anchored with S1R through a salt bridge with residue Glu172; this interaction was further reinforced by hydrogen bond interaction with Glu172.

An additional polar interaction was observed between ionized nitrogen atom and Asp126 for compounds 1, 3 and 4. Furthermore, the two aromatic rings of each ligand created the required hydrophobic interactions with the two sub-pockets; when a fluorine atom and/or hydroxyl-group were placed on phenyl rings, as for ligands 2, 4, and 5, we detected the loss of this multiple interaction with Glu172 mediated by electrostatic and hydrogen binding and/or ionic contact with Asp126 thus leading lower affinity than parent compound 1 and 3. These findings were in good agreement with binding information from X-ray derived structure of S1R co-crystallized 4-IBP (*K*_i_S1R__ 1.7 nM) (Fig. 5F) especially for the polar contacts with crucial residues (Asp126 and Glu172).

To evaluate the stability and the frequency of the interactions for the ligands 1–5 in complex with S1R, we performed molecular dynamic simulations by means the program Desmond of the Schrödinger Suite Desmond.^29,30^

Our investigation was focused on compounds 1 and 5 that resulted the best and poor ligands possessing *K*_i_ values of 3.2 and 434 nM, respectively; therefore, they have been chosen to describe the interactions and the stability. The data corroborated the binding mode observed in the docking results, in which the two ligands were placed in an opposite orientation of the aromatic rings occupying the primary and secondary region.

**Fig. 6:**
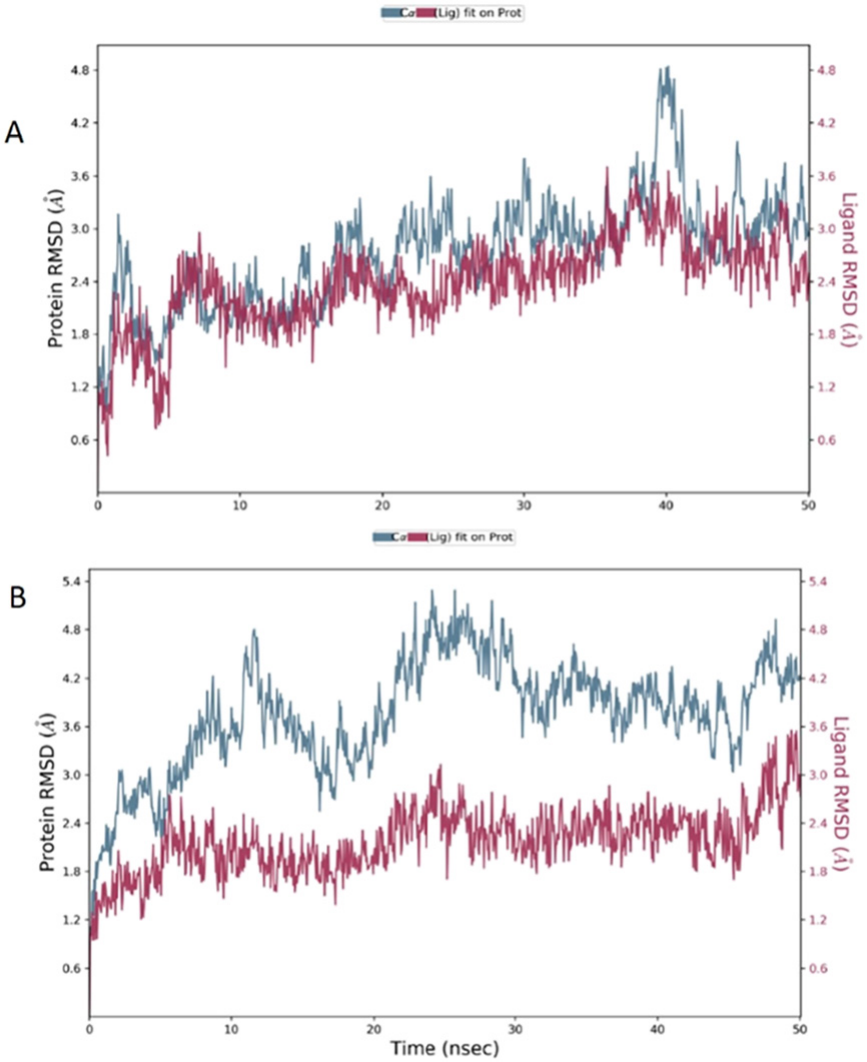
The RMSD plot of the S1R in complex with compounds 1 (A) and 5 (B). In the *X*-axis is reported the simulation time; in the left *Y*-axis is reported the S1R RMSD evolution, while the right *Y*-axis indicates how stable are the compounds in the binding site during the simulation.

The root mean square deviation (RMSD) was calculated during 50 ns simulation and showed the stability of the complex compound 1-S1R during the time evolution; only a small increase in the protein RMSD (blue plot) is observed in the range 38–41 ns. The magenta plot indicated the RMSD evolution of the ligand with respect to the protein and in its cavity, demonstrating the stability of compound 1 in the binding site during the simulation. Despite the complex compound 5-S1R is overall stable, the RMSD data revealed conformational changes in the protein (blue plot) with respect to the reference position at the time 0 (Fig. 6).

**Fig. 7:**
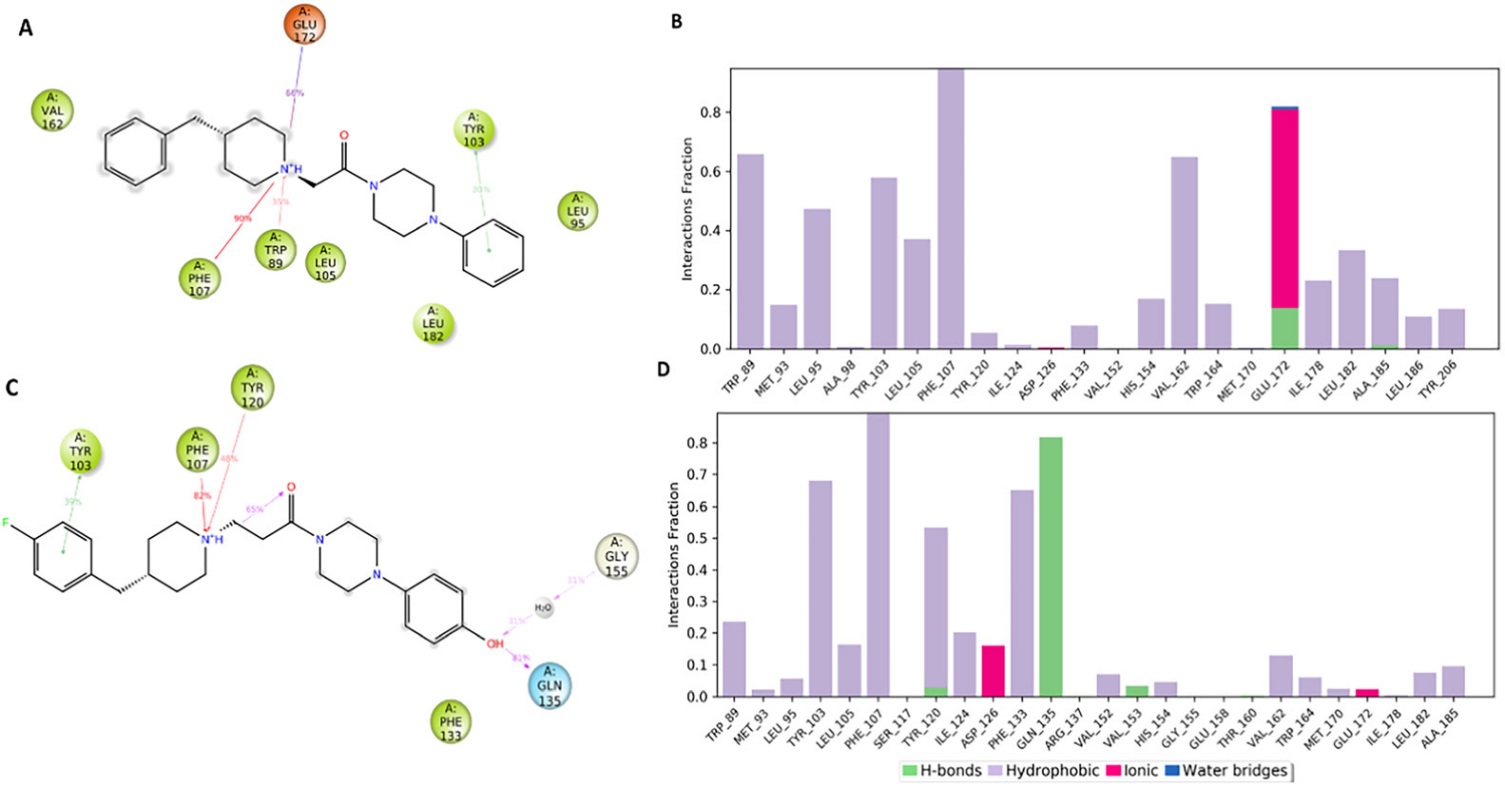
Interactions of compound 1 (panel A) and compound 5 (panel C) with S1R occurring during the MD simulation considering the interactions that are manifested more than 30%. The tables in panel B and panel D show the protein–ligand interactions categorized into four types: hydrogen bonds (green bar), hydrophobic (purple bar), ionic (fuchsia bar) and water bridges (blue bar). A detailed view of the contacts between ligand atoms and residues.

Fig. 7A and B pointed out the specific subtypes of contacts that the detailed atom of the ligand establishes with the residues, considering the contacts that occur more than 30%. The histograms in Fig. 7B and D summarize the total of contacts that the protein established with the ligand during the time simulation. Each bar reports a value that has been converted from a percentage rate to a decimal number. Therefore, a value of 0.5 refers to an interaction kept for the 50% of the time simulation.

Interestingly, only the basic amino moiety of the compound 1 is involved in the essential ionic interaction with the side chain of the Glu172 (66%), supporting our docking data and rationalizing the higher affinity of compound 1 in comparison with 5. Moreover, the ionized amino moiety was engaged in a hydrogen bonding interaction with Glu172 (14%) and a π–cation interaction with Trp89 (35%), enhancing the binding properties of the compound 1. Both the compounds formed a π–cation contact with the aromatic ring of the Phe107 for the whole-time simulation and about 30–39% binding rate with Tyr103 through π–π interactions, indicating an important role played in the binding to the cavity. Regarding the hydrophobic contacts, the plot reports a higher number and frequency of interactions for the compound 1 (panel A). Specifically, the most widely maintained contacts include the residues Trp89 (66%) Leu95 (47%), Tyr103 (58%), Leu105 (37%), Val162 (65%) and Leu182 (34%).

Compound 5 (panel B) was involved in a π–cation interaction with Tyr120 (48%) and in van der Waals contacts with Phe133 (65%). It is important to remark that, although the phenolic portion established a hydrogen bond with Gln135, it unfavourably affected the formation of interactions with the amino acids forming the primary hydrophobic pocket. These results provided a plausible explanation about the different degree of binding affinity measured by *in vitro* binding assays for compounds 1 and 5.

Overall, the molecular modelling studies suggested that a fruitful strategy to improve S1R affinity should involve additional contacts directed to the two opposite pockets hosting primary and secondary hydrophobic groups of potent ligands. Moreover, the design of new S1R ligands has to enrol the basic amino moiety as a pharmacophoric requirement for locking the crucial multi-polar interaction with crucial residue Glu172.

## Experimental

### Chemistry

The chemical collection of already studied compounds that are available in our laboratory was the source of compounds 1–13 for the primary screening. For all tested compounds the purity was ≥95%. For the thirteen molecules the spectral characterization was in good agreement with data reported in previous papers in which the synthetic route is thoroughly described (compounds 1–5,^31^6–9,^32^10–11,^33^ and 12–13 (ref. 34)). In Supporting Information section, we briefly described the synthetic procedure to prepare the most active ligand for functional assay.

### Radioligand binding assays

S1R and S2R radioligand binding assays involve the use of liver homogenates from male Sprague Dawley rats as previously reported.^21,22^*In vitro* S1R ligand binding assays were performed in Tris buffer (50 mM, pH 8), with [^3^H](+)-pentazocine (2 nM) as radioligand. The final volume was 0.5 mL. The *K*_d_ value of [^3^H](+)-pentazocine was 2.9 nM. Measurement of non-specific binding was carried out using unlabeled (+)-pentazocine (10 μM). *In vitro* S2R ligand binding assays were performed in Tris buffer (50 mM, pH 8.0), using [^3^H]DTG (2 nM) as radioligand. (+)-Pentazocine (5 μM) was used as S1R masking agent. The final volume was 0.5 mL. The *K*_d_ value of [^3^H]DTG was 17.9 nM. Measurement of non-specific binding was carried out using DTG (10 μM).

### S1R functional assay

Functional profile of compound 1 was obtained by using the phenytoin method which uses the same protocol as S1R radioligand binding assay with some differences. The experiments were performed one time in the presence of phenytoin and one time without phenytoin. In both cases the final volume was 0.5 mL and it was composed as follows: 200 μL of membrane preparation, 50 μL of 20 nM [^3^H](+)-pentazocine (28.4 Ci mmol^−1^, PerkinElmer), 50 μL of cold ligand or its solvent, 180 μL of Tris buffer (50 mM, pH 8) and 20 μL of 25 mM phenytoin (Merck Life Science S.r.l.) or its solvent (0.3 M NaOH). The incubation of samples lasts 120 min at 37 °C. Unlabeled (+)-pentazocine (10 μM) was used to measure non-specific binding. The molecules are defined S1R agonist if the *K*_i_ ratio without/with phenytoin is >1 and antagonist if the *K*_i_ ratio without/with phenytoin is ≤1.^24^

### Functional assay data analysis

GraphPad Prism® 7.0 (GraphPad Software, San Diego, CA, USA) program was used to calculate the *K*_i_ values that are given as mean value ± SD from at least two independent experiments performed in duplicate.

### Computational studies

Computational studies were performed using the software Maestro (Schrödinger Release 2020-4: Maestro, Schrödinger, LLC, New York, NY. 2020) on both the compounds 1–11 and the co-crystalized ligands. The 2D structures of our library were built by means the 2D Sketcher tool and then converted in 3D structures. All the ligands were prepared by means LigPrep implemented in the Schrödinger suite (Schrödinger release 2020-4: LigPrep, Schrödinger, LLC, New York, NY. 2020), setting up a pH value of 7.4. To retrieve the main state at pH that mimic the physiological one, OPLS4 as force field and keeping the original configuration for each molecule containing chiral centers. The 3D crystallographic structure of S1R in complex with the ligand 4-IBP was used as model protein, available on the Protein Data Bank (PDB ID: 5HK2).^13^ Firstly, the co-crystallized ligand and the waters molecules were deleted and then the protein was submitted to a preparation protocol using the Protein Preparation Wizard module in Maestro software.^35^ Specifically, the hydrogens have been added, the bond orders have been assigned, and the missing side chains were filled. Therefore, the protein was submitted to a minimization to relieve tension, optimize the structure, and adjust the position of the various groups. Rigid docking was computed by means the Ligand Docking module of Glide.^26^ The grid box was built using the Receptor Grid Generation tool,^26^ identifying the 4-IBP as centroid and a size of 15 Å from the ligand. The default parameter Van der Waals radius scaling was applied, with scaling factor of 1.0 and partial charge cut-off 0.25 and no constraints have been defined. The calculation was performed using SP (Standard Precision) method, the OPLS4 force field and default Van der Waals scaling factor, with scaling factor of 0.80 and partial charge cut-off 0.15. For each ligand were reported 10 poses and the one with the best GlideEmodel score was retained for the next phase. The induced fit docking (IFD)^36^ protocol takes in account the flexibility of the side chain and comprises the following steps: Glide Docking, Prime Refinement^37^ and Glide Redocking. We set up the best pose selected from the rigid docking as centroid and the optimization of the side chain, performed by the tool Prime, included the residues within 5 Å of the ligand pose. The SP method, the Van de Waals scaling factor and the number of reported poses parameters are the same employed in the ligand docking procedure. The top ranked Gscore pose was selected for the further analysis.

The molecular dynamics (MD) simulations on the IFD complex were carried out by means the tool Desmond^29,30^ of the Schrödinger Suite. The complex was placed in a box with an orthorhombic shape and a size of 10, 10, 16 Å in the *x*, *y* and *z* directions. The TIP3P was used as solvent model, the salt NaCl was adding at a concentration of 0.15 M and the system was neutralized by adding Na ions and the OPLS4 was used as force field. The POPC was selected as membrane model and then was set up at the level of the transmembrane alpha helix including the residues 10–31; the placement of the membrane was performed trough the OPM (Orientations of Proteins in Membranes) convention. MD simulations were performed for 50 ns in the NPT ensemble, specifically at 1 atm pressure and 300 K temperature.

## Conclusions

In summary, these investigations allowed us to identify the prototype compound 1, which was able to efficiently bind the S1R and resulted equipotent with reference compound haloperidol. Noteworthy, in the phenytoin functional assay, compound 1 emerged as S1R agonist. The network of interaction with S1R was elucidated through docking analysis as well as molecular dynamic simulations. From these studies we suggested that compound 1 assumed similar pharmacophoric elements when compared to well-known S1R ligands haloperidol, 4-IBP, and PD144418. Furthermore, we identified selective S1R ligands even if the binding pockets of the S1R and S2R are very similar. These data confirmed the crucial role of polar as well as hydrophobic interactions to stabilize the ligands within the receptors sub-pockets; these results would guide our future design to identify potent S1R ligands.

## Author contributions

Conceptualization: R. G. and L. D. L. Funding acquisition: R. G. and E. A. Investigation: L. L.; S. M., M. D., G. C. Validation: L. D. L., E. A., R. G. and A. M. R.G. and L. D. L. wrote the manuscript with input from all authors. All authors have read and agreed to the published version of the manuscript.

## Conflicts of interest

There are no conflicts to declare.

## Supplementary Material

MD-014-D3MD00291H-s001
