# Supplementary material for: Discovery and computational studies of piperidine/piperazine-based compounds endowed with sigma receptor affinity
Source: RSC Med Chem. 2023 Jul 26;14(9):1734–42. doi: 10.1039/d3md00291h (PMC10507793; doi:10.1039/d3md00291h)
Supplement: MD-014-D3MD00291H-s001 [file MD-014-D3MD00291H-s001.pdf]

Electronic Supplementary Material (ESI) for RSC Medicinal Chemistry.
This journal is © The Royal Society of Chemistry 2023

Supplementary Information

Discovery and computational studies of piperidine/piperazine-based compounds endowed with sigma receptor affinity

Laura De Luca,[a] Lisa Lombardo,[a] Salvatore Mirabile,[a] Agostino Marrazzo,[b] Maria Dichiara,[b] Giuseppe Cosentino,[b] Emanuele Amata,[b] Rosaria Gittoa,*[a]

[a]Dipartimento di Scienze Chimiche, Biologiche, Farmaceutiche ed Ambientali, Università degli Studi di Messina, Viale Ferdinando d'Alcontres 31, 98166 Messina, Italy
[b]Dipartimento di Scienze del Farmaco e della Salute, Università degli Studi di Catania, Viale Andrea Doria 6, 95125 Catania, Italy.

Contents

1. **Synthetic procedure and ^{1}H/^{13}C NMR data for the most interesting and known compound 1**
2. **Computational protocol validation**

1. Synthetic procedure and ^{1}H/^{13}C NMR data for the most interesting and known compound 1

Compound **1** was synthesized according to the previously reported procedure.[1] All reagents and solvents were obtained from commercial suppliers (Sigma-Aldrich, Milan, Italy) and used without further purification. NMR spectra were acquired with a Varian Gemini 500 spectrometer (Palo Alto, CA, USA).

Preparation of 2-[4-(benzyl)piperidin-1-yl]-1-(4-phenylpiperazin-1-yl)ethanone (1)

1-Phenylpiperazine (425 mg, 2.62 mmol) was solubilized in DCM (4 mL) and, then, chloroacetyl chloride (2.62 mmol, 208.7 µl) was added slowly (0 °C). The reaction mixture was stirred at room temperature for 1 hour. A saturated solution of NaHCO3 (5 mL) was added to quench the reaction. The mixture was extracted with DCM twice and the solvent was removed under vacuum. The desired intermediate was crystalized with Et_2O. Subsequently, to a solution of the obtained 2-Chloro-1-(4-phenylpiperazin-1-yl)ethanone (240 mg, 1.0 mmol) in DMF (4 mL) the 4-benzylpiperidine (1.5 mmol, 263.7µl) and K_2CO_3 (69 mg, 0.5 mmol) were added. The reaction was carried out in microwave for 15 min at 100 °C and then was quenched with water (5 mL) and a saturated solution of $NaHCO_3$ (5 mL). The aqueous layer was extracted with EtOAc (3x10 mL) and organic phases were washed with brine, dried over Na_2SO_4, concentrated and finally treated with EtOH and Et_2O to afford the desired compound **1**.

Characterization of 2-Chloro-1-(4-phenylpiperazin-1-yl)ethanone (intermediate)

Yield: 58%. White powder. M.p.: 73–75 °C. ^{1}H-NMR (500 MHz, DMSO-d_6): (δ) 3.38 (d, *J*=26.3 Hz, 4H, 2CH_2), 3.82 (bs, 4H, 2CH_2), 4.48 (s, 2H, CH_2), 7.15 (bs, 1H, ArH), 6.41 (m, 4H, ArH,). Anal. Calculated for ($C_{12}H_{15}ClN_2O$): C 60.38, H 6.33, N 11.74. Found: C 60.43, H 6.30, N 11.78.

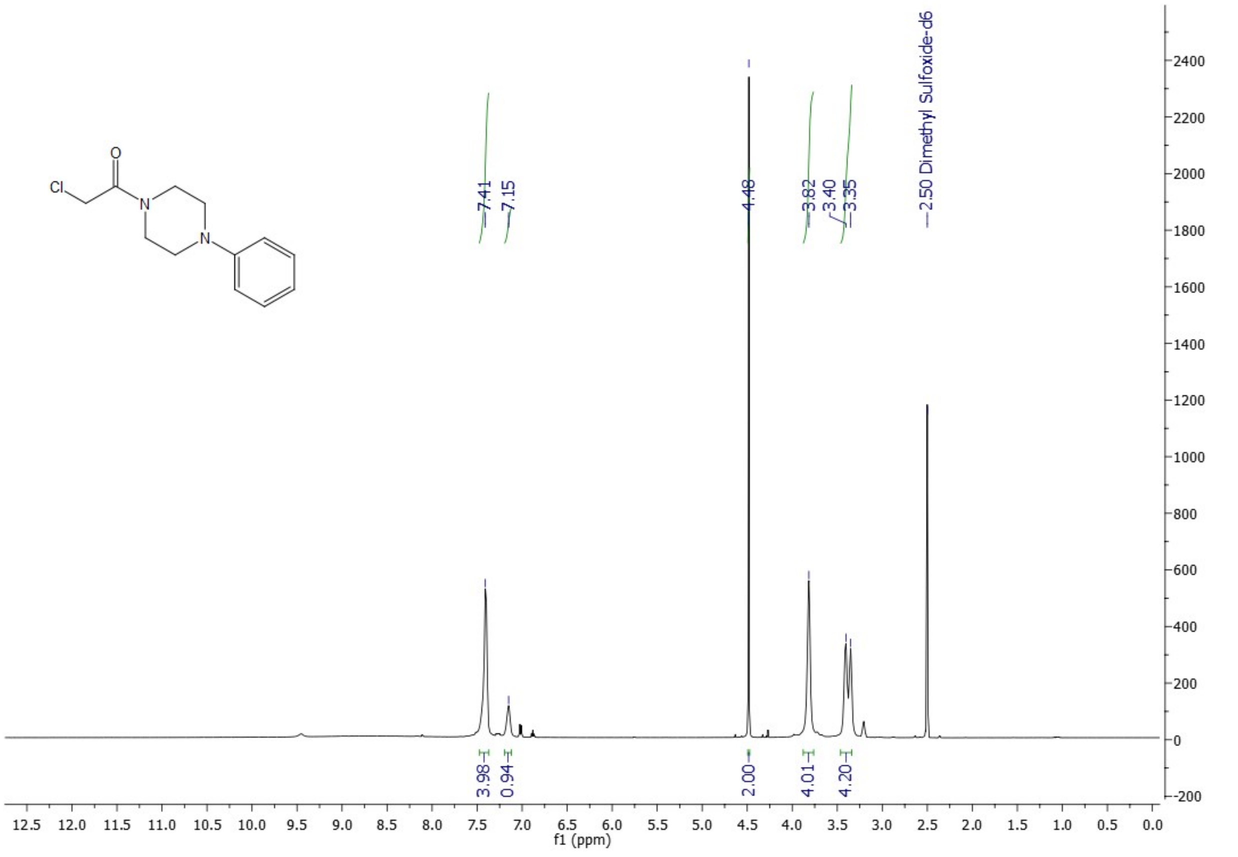

Fig. S1. ^{1}H-NMR (DMSO-d_6) spectrum of 2-Chloro-1-(4-phenylpiperazin-1-yl)ethanone.

Characterization of 2-[4-(benzyl)piperidin-1-yl]-1-(4-phenylpiperazin-1-yl)ethanone (1)

Yield: 46%. White powder. M.p.: 94–96 °C. ^{1}H-NMR (500 MHz, DMSO-d_6): (δ) 1.17 (m, 2H, CH_2), 1.45 (bs, 1H, CH), 1.52 (d, *J*= 12.3 Hz, 2H, CH_2), 1.91 (t, *J*= 11.0 Hz, 2H, CH_2), 2.48 (d, *J*= 7.3 Hz, 2H, CH_2), 2.78 (d, *J*= 11.4 Hz, 2H, CH_2), 3.06 (m, 2H, CH_2), 3.13 (m, 2H, CH_2), 3.57 (m, 2H, CH_2), 3.69 (m, 2H, CH_2), 6.81 (m, 1H, ArH), 6.96 (d, *J*= 8.0 Hz, 2H, ArH), 7.16 (m, 3H, ArH), 7.24 (m, 4H, ArH). ^{13}C-NMR (126 MHz, DMSO-d_6): 31.7, 37.0, 41.0, 42.3, 45.0, 48.4, 49.0, 53.0, 61.4, 115.8, 119.2, 125.7, 128.1, 129.9, 140.3, 150.9, 167.7. Anal. Calculated for ($C_{24}H_{31}N_3O$): C 76.35, H 8.28, N 11.13. Found: C 76.41, H 8.25, N 11.16.

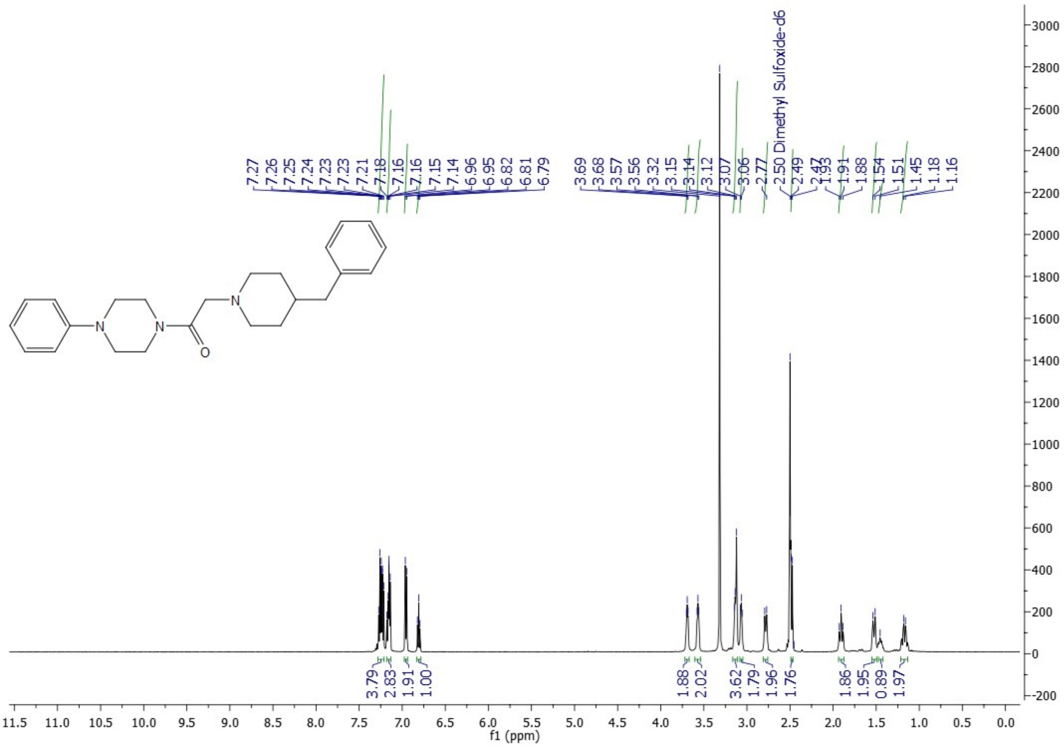

Fig. S2. ^{1}H-NMR (DMSO-d_6) spectrum of 2-[4-(benzyl)piperidin-1-yl]-1-(4-phenylpiperazin-1-yl)ethanone (**1**)

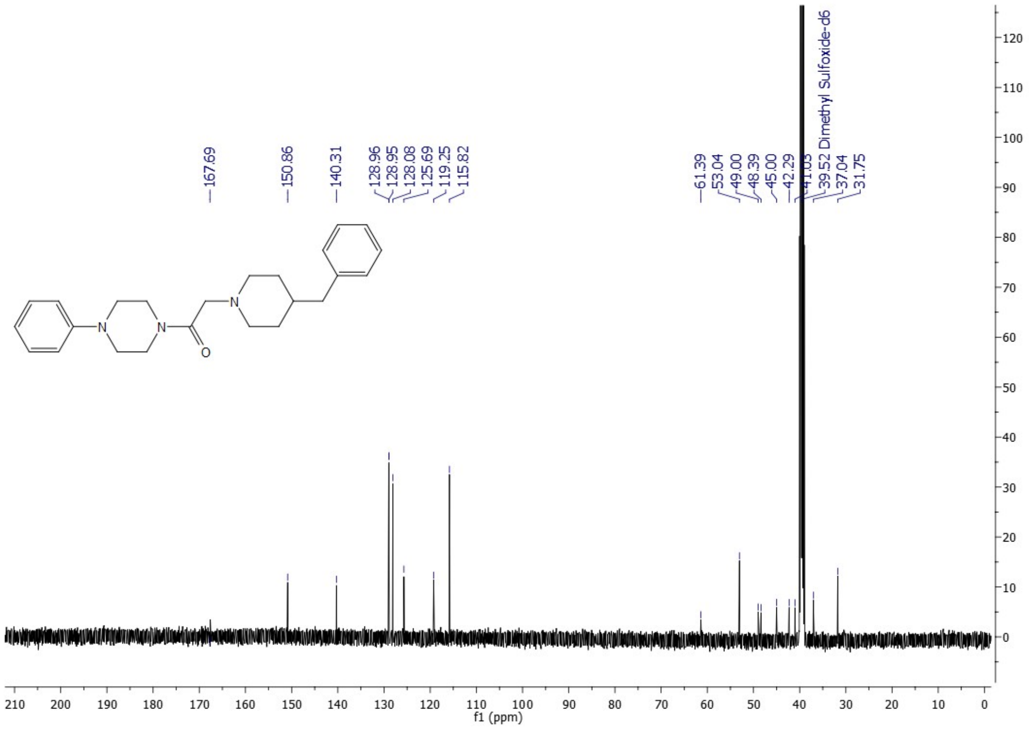

Fig. S3. ^{13}C-NMR (DMSO-d_6) spectrum of 2-[4-(benzyl)piperidin-1-yl]-1-(4-phenylpiperazin-1-yl)ethanone (**1**)

2. Computational protocol validation

2.1 To obtain a predictive computational protocol, we submitted the co-crystalized ligands 4-IBP, PD144418 and haloperidol (PDB codes: 5HK2, 5HK1 and 6DJZ respectively) to the following workflow using the software Maestro (Schrödinger Release 2020-4: Maestro, Schrödinger, LLC, New York, NY. 2020): rigid docking, flexible docking and molecular dynamic simulations.

Once the rigid docking was carried out, the best glide Emodel pose selected for each ligand was subsequently overlapped to the crystallographic one. The RMSD values confirmed the predictivity of our protocol, showing a RMSD of 0.7153 Å, 0.5773 Å and 1.0357 Å between the binding pose and the crystalized one of respectively 4-IBP (Fig. S4A), PD144418 (Fig. S4C) and haloperidol (Fig. S4E). Therefore, the best pose for each ligand has been subjected to a redocking calculation considering the flexibility of the side chain by means the Induced Fit Protocol (IFD) of the Schrodinger Suite. Again, the superimposition of the best pose (Glide Gscore fitness score) respect to the crystalized one corroborate the predictivity of the protocol. In detail, the RMSD calculated between the IFD poses and the crystallographic ligands were 0.9854 Å for 4-IBP (Fig. S4B), 0.7374 Å for PD144418 (Fig. S4D) and 1.7209 Å for haloperidol (Fig. S4F). The bigger values obtained for haloperidol may be attributed to the more flexibility of butil-4-one linker; overall, the pose keeps a conserved binding mode respect to the crystallographic data.

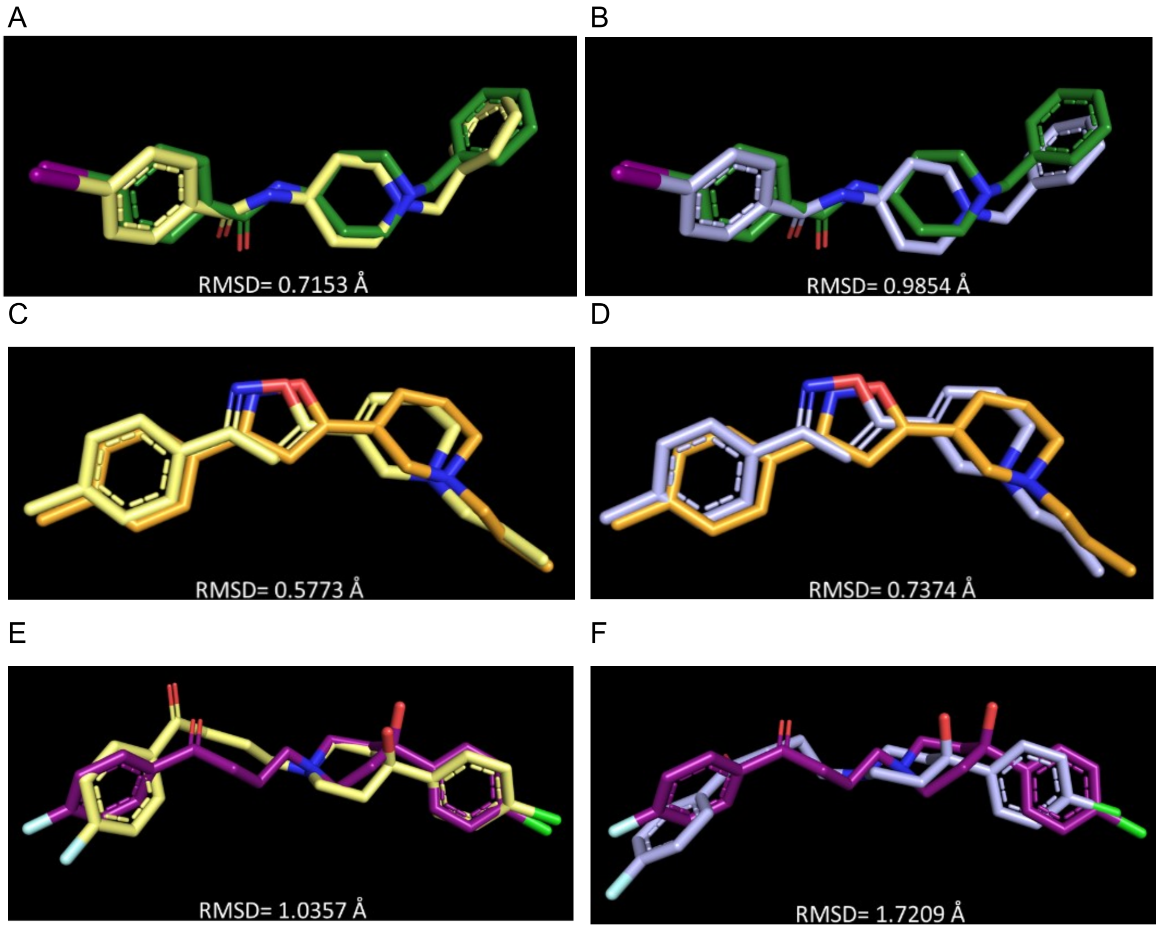

Fig. S4. (A) Superimposition of the co-crystalized 4-IBP (green sticks) on the rigid docking (pale yellow sticks). (B) Superimposition of the co-crystalized 4-IBP (green sticks) and induced Fit Docking poses (violet sticks). (C) Superimposition of the co-crystalized PD144418 (orange sticks) on the rigid docking (pale yellow sticks). (D) Superimposition of the co-crystalized PD144418 (orange sticks) on the induced Fit Docking poses (violet sticks). (E) Superimposition of the co-crystalized haloperidol (purple sticks) on the rigid docking (pale yellow sticks). (F) Superimposition of the co-crystalized haloperidol (purple sticks) on the induced Fit Docking poses (violet sticks).

2.2 To obtain a procedure with high accuracy, the protocol validation comprises the molecular dynamics simulations on the complex selected from Induced Fit Docking. The plots below show the RMSD evolution of the S1R in complex with the ligand 4-IBP (Fig. S5A), PD144418 (Fig. S6A) and haloperidol (Fig. S7A). The protein RMSD values (left Y-axis) are calculated based on the reference frame at the time 0 indicating possible conformational changes during the 50 ns simulation (X-axis). The increase of the RMSD of the S1R in the three simulations may be attributed to the tail N-terminal (1-10 AA) as reporting in the RMSF (The Root Mean Square Fluctuation) that characterize local changes along the protein chain. The peaks depicted in the RMSF plots (Fig. 5B, 6B and 7B) point out areas of the protein that fluctuate the most during the simulation, while green vertical bars marked the residues interacting with the ligand. Considering the collected data, the protein structures are overall equilibrated during the simulation.

The Ligands RMSD (right Y-axis) indicates that the three crystallographic compounds are stables respect to the protein and its binding pocket, showing RMSD values around 2 Å.

A

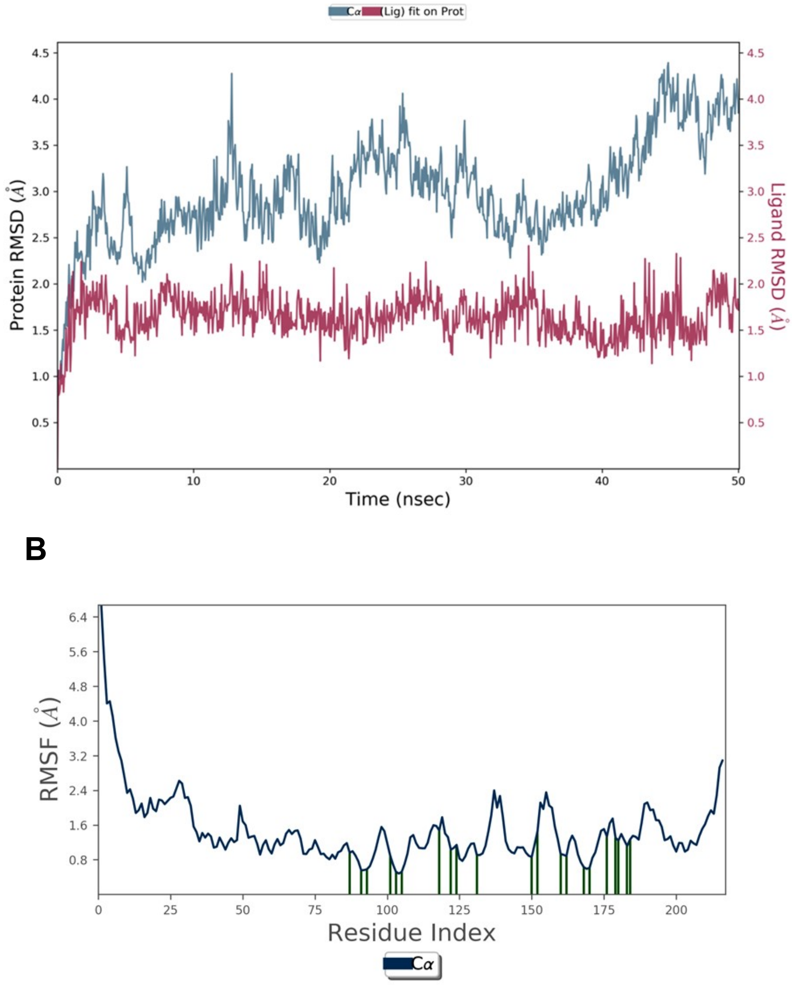

Fig. S5. (A) The RMSD plot of the 4-IBP in complex with S1R. The blue plot indicates the RMSD protein evolution during the simulation time (X-axis), whose values are reported in the left Y-axis; the magenta plot represents the RMSD of the ligand respect to the protein and the correlated values are showed in the right Y-axis. (B) The RSMF plot reports the fluctuations of the protein chain highlighted by the peaks. The green bars point out the residues interacting with the ligand.

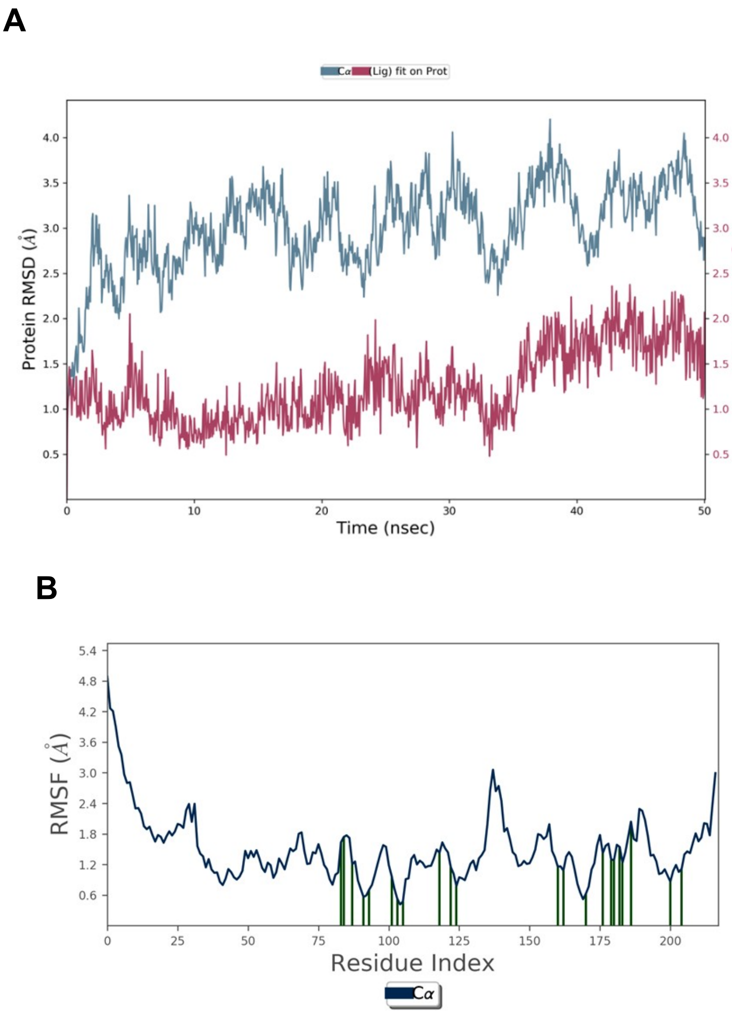

Fig. S6. (A) The RMSD plot of the PD144418 in complex with S1R. The blue plot indicates the RMSD protein evolution during the simulation time (X-axis), whose values are reported in the left Y-axis; the magenta plot represents the RMSD of the ligand respect to the protein and the correlated values are showed in the right Y-axis. (B) The RSMF plot reports the fluctuations of the protein chain highlighted by the peaks. The green bars point out the residues interacting with the ligand.

A

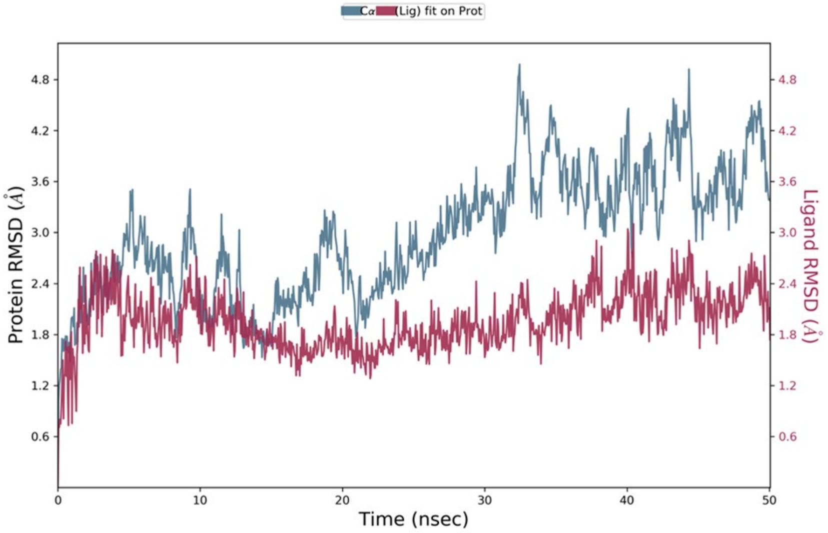

B

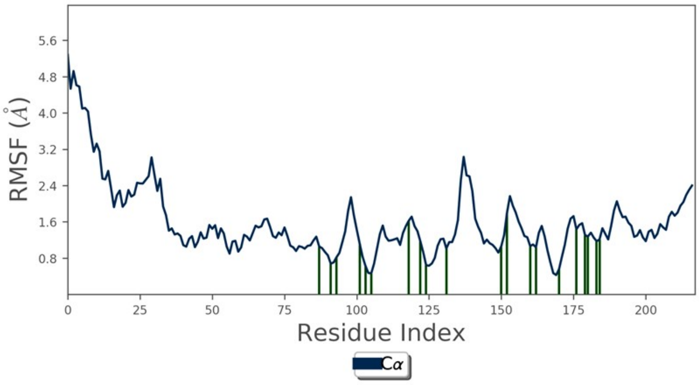

Fig.S7. (A) The RMSD plot of the haloperidol in complex with S1R. The blue plot indicates the RMSD protein evolution during the simulation time (X-axis), whose values are reported in the left Y-axis; the magenta plot represents the RMSD of the ligand respect to the protein and the correlated values are showed in the right Y-axis. (B) The RSMF plot reports the fluctuations of the protein chain highlighted by the peaks. The green bars point out the residues interacting with the ligand.

The contacts established during the simulations are summarizes in the histograms (Fig. S8A, S9A and S10A). Each bar is referred to a specific residue interacting with ligands by means one or multiple type of interactions. The values expressed in decimal number describe the frequency of the interaction kept during the time simulation. Therefore, a value of 0.5 refers to an interaction kept for the 50% of the time simulation. Moreover, values over 1.0 are possible as some residues may be involved in multiple interactions of same type with different functional groups or atoms of the ligand. It is important to note that the ionic interaction (magenta bar) between two oppositely charged atoms are reported when they are not involved in a hydrogen bond simultaneously. As a result, despite the ionic contacts between Glu172 and 4-IBP as well as PD144418 is established during the entire simulation time, the plot reported it for a small range. Lasty, the Figure 8, 9 and 10 B display a graphic of the specified ligand atom interacting with the residues, considering the contact occur more than 30.0% of the simulation time.

Taking in account these notions, the obtained data are consistent with the experimental one since all compounds are able to form the essential interactions by means the basic centre and the two flanked hydrophobic moieties as reported in literature.[2, 3] Overall, these results indicate the strength of our protocol.

A

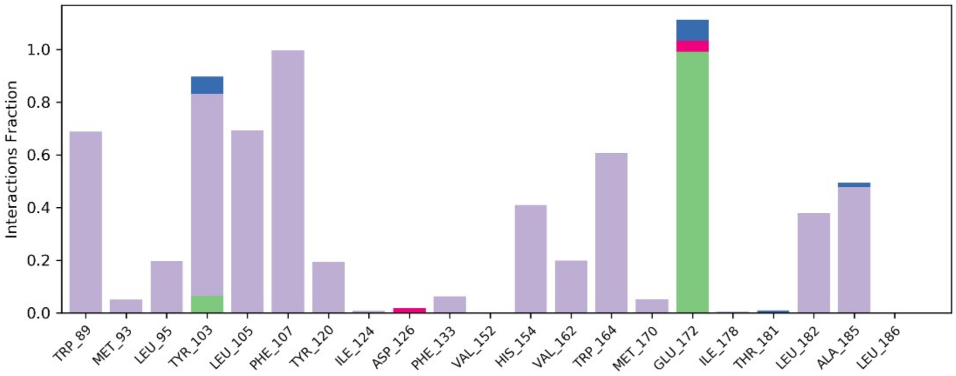

B

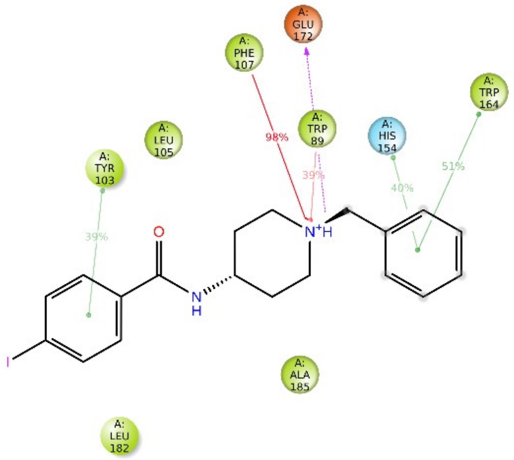

Fig. S8. The plots of the interactions between compound 4-IBP and S1R occurring during the simulation. (A) The table shows the protein-ligand interactions categorized into four types: Hydrogen Bonds (green bar), Hydrophobic (purple bar), Ionic (fuchsia bar) and Water Bridges (blue bar). (B) A detailed view of the contacts between ligand atoms and residues, considering the interactions that are manifested more than 30%.

A

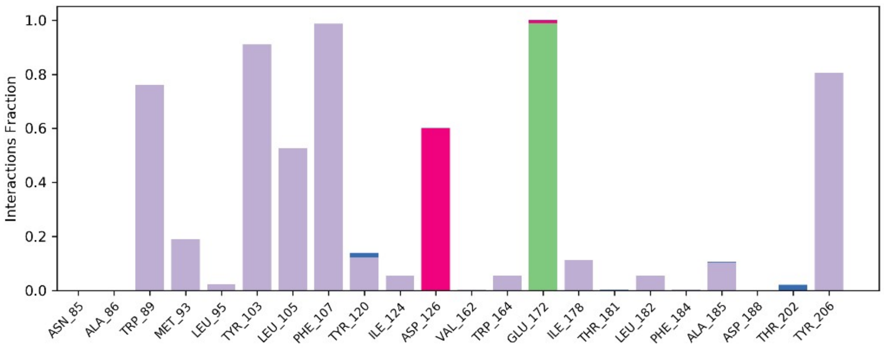

B

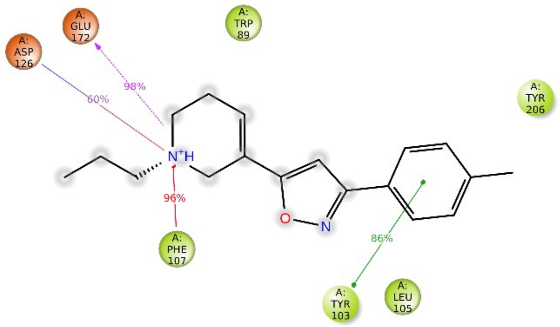

Fig. S9. The plots of the interactions between compound PD144418 and S1R occurring during the simulation. (A) The table shows the protein-ligand interactions categorized into four types: Hydrogen Bonds (green bar), Hydrophobic (purple bar), Ionic (fuchsia bar) and Water Bridges (blue bar). (B) A detailed view of the contacts between ligand atoms and residues, considering the interactions that are manifested more than 30%.

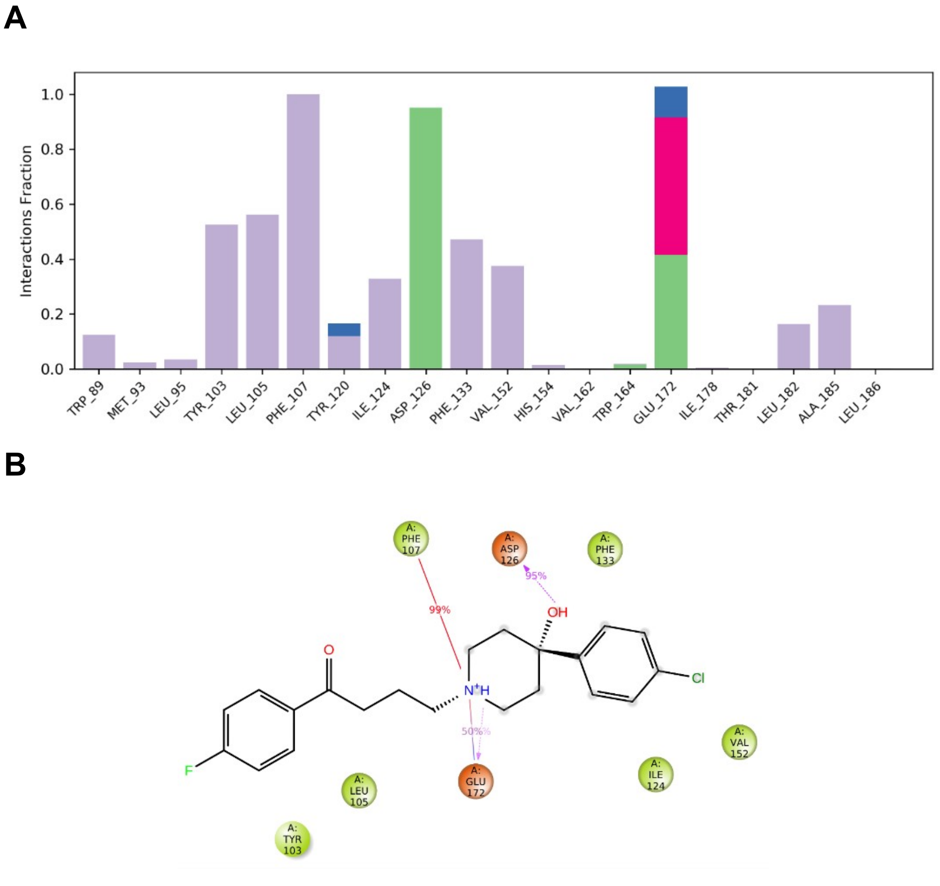

Fig. S10. The plots of the interactions between compound haloperidol and S1R occurring during the simulation. (A) The table shows the protein-ligand interactions categorized into four types: Hydrogen Bonds (green bar), Hydrophobic (purple bar), Ionic (fuchsia bar) and Water Bridges (blue bar). (B) A detailed view of the contacts between ligand atoms and residues, considering the interactions that are manifested more than 30%.

REFERENCES

1. De Luca, L.; Germanò, M. P.; Fais, A.; Pintus, F.; Buemi, M. R.; Vittorio, S.; Mirabile, S.; Rapisarda, A.; Gitto, R. Discovery of a new potent inhibitor of mushroom tyrosinase (Agaricus bisporus) containing 4-(4-hydroxyphenyl)piperazin-1-yl moiety. *Bioorg Med Chem* **2020**, *28* (11), 115497.

2. Schmidt, H. R.; Zheng, S.; Gurpinar, E.; Koehl, A.; Manglik, A.; Kruse, A. C. Crystal structure of the human σ1 receptor. *Nature* **2016**, *532* (7600), 527-530.

3. Schmidt, H. R.; Betz, R. M.; Dror, R. O.; Kruse, A. C. Structural basis for σ. *Nat Struct Mol Biol* **2018**, *25* (10), 981-987.
